# Astrocytes are direct cellular targets of lithium treatment: novel roles for lysyl oxidase and peroxisome-proliferator activated receptor-γ as astroglial targets of lithium

**DOI:** 10.1038/s41398-019-0542-2

**Published:** 2019-09-02

**Authors:** Andrea D. Rivera, Arthur M. Butt

**Affiliations:** Institute of Biomedical and Biomolecular Sciences, School of Pharmacy and Biomedical Science, University of Portsmouth, St Michael’s Building, White Swan Road, Portsmouth, PO1 2DT UK

**Keywords:** Molecular neuroscience, Bipolar disorder

## Abstract

Astrocytes are multifunctional glial cells that play essential roles in supporting synaptic signalling and white matter-associated connectivity. There is increasing evidence that astrocyte dysfunction is involved in several brain disorders, including bipolar disorder (BD), depression and schizophrenia. The mood stabiliser lithium is a frontline treatment for BD, but the mechanisms of action remain unclear. Here, we demonstrate that astrocytes are direct targets of lithium and identify unique astroglial transcriptional networks that regulate specific molecular changes in astrocytes associated with BD and schizophrenia, together with Alzheimer’s disease (AD). Using pharmacogenomic analyses, we identified novel roles for the extracellular matrix (ECM) regulatory enzyme lysyl oxidase (LOX) and peroxisome proliferator-activated receptor gamma (PPAR-γ) as profound regulators of astrocyte morphogenesis. This study unravels new pathophysiological mechanisms in astrocytes that have potential as novel biomarkers and potential therapeutic targets for regulating astroglial responses in diverse neurological disorders.

## Introduction

Lithium has been in continuous use as a mood stabiliser in the treatment of bipolar disorder (BD) and major depression for decades, but its precise mode of action remains unclear^1^. Pharmacological and genetic studies have identified a number of key cellular signalling pathways that are effected by lithium, among the most prominent being glycogen synthase kinase 3 (GSK3), cAMP response element-binding protein (CREB) and inositol^1^. In addition, transcriptomic methods are identifying lithium-responsive genes that are beginning to provide compelling links with neurobiological studies^2^. At a cellular level, lithium is neuroprotective and, as the primary neuroprotective cells of the CNS, there is increasing interest in astrocytes being prominently involved in mood disorders and potential targets of lithium^3^.

Astrocytes perform essential structural and homoeostatic functions that maintain neuronal signalling and integrity^4^. Disruption of astroglial functions has critical consequences for cognitive function implicated in neuropsychiatric disorders and dementia, including BD, schizophrenia and Alzheimer’s disease (AD)^5–8^. The morphological and physiological changes that astrocytes undergo in response to pathology is generally termed ‘astrogliosis’ or ‘reactive astrogliosis’, most often characterised by an upregulation of the astrocyte intermediate filament glial fibrillary acidic protein (GFAP) and cellular hypertrophy, which may or may not be associated with cell proliferation^4^. However, reactive astrogliosis is not uniform and varies in a context-specific manner, with two extremes of the spectrum being reactive astrogliosis that is either deliterious or beneficial for protection and repair^9,10^. In BD, deficiencies in astroglial function are implicated in changes in synaptic signalling and these changes may be modified by anti-bipolar drugs, including lithium^11^. Notably, specifically targeting astrocytes in rodents induces neuropathology and behavioural changes characteristic of depression, schizophrenia and BD^12^. Astrocyte changes have been detected by post-mortem analysis of the astrocyte-specific protein GFAP in BD, schizophrenia and AD^13–15^. In addition, mRNA levels of GFAP have been shown to be increased in the peripheral blood of BD subjects never treated with lithium, compared to BD subjects treated continuously with lithium, which had similar levels of GFAP as control subjects^16^. Together, these studies raise the possibility that the effects of lithium on astrocytes may be related to its beneficial therapeutic effects.

As well as being the frontline treatment for BD, lithium is also effective as an add-on medication in schizophrenia^17^ and has beneficial effects on cognitive performance in subjects with AD^18^. There is growing evidence that lithium positively regulates astroglial pathology in disease models of AD^19^, as well as Alexander disease^20^ and Fragile-X syndrome^21^. The majority of studies on astrocytes focus on their role in maintaining synaptic signalling in grey matter (GM). However, communication between GM regions depends on white matter (WM), comprised of bundles of myelinated axons that are essential for the superfast connectivity of the human brain, and WM disruption is implicated in all neuropsychiatric diseases including BD^22,23^. Astrocytes are essential for WM function and integrity^24^, and there is clear evidence in prefrontal WM of disruption of astrocytes and axons in BD^25^. Moreover, lithium preferentially accumulates in WM and counteracts disruption of WM in BD^23^. This led us to propose that WM astrocytes may be direct cellular and molecular targets of lithium. Here, we have tested this directly in situ in the adult mouse optic nerve, a model WM tract that contains only glial cells and the axons they support^24^. The results demonstrate for the first time that the ECM-regulatory enzyme lysyl oxidase (LOX) is a novel astroglial target of lithium and a profound regulator of astrocyte morphology and proliferation. Moreover, pharmacogenomic analysis identified drugs that target peroxisome proliferator-activated receptor gamma (PPAR-γ) regulate astrogliosis and have therapeutic potential in diverse neurodegenerative and neuropsychiatric disorders.

## Materials and methods

### Animals and tissue

Young adult mice aged 35–45 days were used throughout, either transgenic mice in which the astrocyte gene GFAP drives expression of the fluorescent reporter enhanced green fluorescent protein (EGFP)^26^, or the wildtype background mouse strain C57/BL10. All animals were killed humanely in accordance with the Animals Scientific Procedures Act (1984).

### Mouse optic nerve culture

The ex vivo mouse optic nerve model was established in our laboratory^27^ and was modified as follows. Optic nerves were removed with the retina intact and placed immediately in ice-chilled oxygenated artificial (a)CSF composed of: NaCl 133 mM, KCl 3 mM, CaCl_2_ 1.5 mM, NaH_2_PO_4_ 1.2 mM, HEPES buffer 10 mM pH 7.3, 0.5% penicillin and streptomycin (Invitrogen); *n* = 6 optic nerves from 3 mice were used per experimental group for confocal microscopy analysis, and *n* = 12 nerves from 6 mice were used for transcriptomic analysis, according to power calculations to ensure sample sizes were adequate to detect statistical differences. Nerves were carefully cleaned of the arachnoid membrane and any attached peripheral tissue, then washed in aCSF and placed on semiporous culture membrane inserts (Millipore 0.4 µm), with 1 ml of medium (consisting of 25% horse serum, 49% OptiMEM, 25% Hanks’s balanced salt solution, 0.5% 25 mM glucose, 0.5% penicillin and streptomycin; all reagents from Invitrogen), and maintained ex vivo in culture at 37 °C in 95%O_2_/5% CO_2_ for 3 days. Lithium chloride (20 mM) was added directly to the culture medium (agents from Sigma Aldrich), based on previously published dose–response curves^27^; the outer integral layer of the optic nerve (pia mater) has a highly restrictive permeability and lithium was added as a stock solution (20 mM) to provide a therapeutic concentration of 1–2 mM in the nerve^27^. After 3 days, the optic nerve tissue was prepared for confocal imaging or RNA extraction. All experiments were conducted in triplicates and no samples were excluded; due to the study design animals were not selected blinded for group allocation, but all outcome measurements were subsequently conducted blind, and all samples were included.

### Confocal microscopy and image analysis

Optic nerves were immersion fixed in 4% paraformalhdehyde (PFA) in phosphate-buffered saline (PBS) for 1 h at RT and following washes in PBS were whole-mounted on microscope slides in VectaShield (VectorLabs). Cells expressing the GFAP-EGFP reporter were visualised at 488 nm using an argon laser and images captured on a Zeiss LSM 710 metaconfocal microscope, using a ×20 Plan-NEOFLUAR 20 objective with a numerical aperture of 0.50. Images were captured maintaining the acquisition parameters constant between samples. Each nerve counted as a single ‘n’ value and the total number of cells was counted midway along the length of the optic nerve in a single field of view (FOV), comprised a constant volume of 200 µm × 200 µm in the *x*–*y*-plane and 25 µm in the *z*-plane, commencing 15 μm below the pial surface. For all comparisons, the significance level was set to 5%; due to the explorative nature of this study, no adjustment was made to the significance level. Cell counts are expressed as mean number of cells per FOV ± standard error of the mean (SEM). There were six nerves from three mice in each experimental group and statistical analysis was performed using GraphPad Prism v3.02 for one-way analysis of variance (ANOVA) followed by Bonferroni post hoc test unless otherwise stated.

### Microarray

As detailed previously^27,28^, maintaining strict RNAase-free and sterile conditions throughout, RNA was extracted and processed using a RNeasy Micro kit (Qiagen). RNA concentration was determined using Nanodrop ND-1000 spectrophotometer and samples were then stored at −80 °C until use. For microarray, RNA was converted to double stranded cDNA and purified, using the Bioarray Single RNA Amplification and Labelling kit and cDNA purification kit (Enzo Life Science). Double stranded cDNA was used to generate multiple copies of biotinylated cRNA using the Bioarray Highyield RNA transcription Labelling Kit (Enzo Life Sciences). Quality control on the biotinylated cRNA produce included the determination of the A260/280 ratio; all samples passed quality control (ATLAS-Biolabs Co., Germany). For microarray chip hybridisation, 10 µg of each biotinylated cRNA sample was hybridised on an Affymetrix GeneChip Mouse Genome 430 2.0 for 16 h at 45 °C, using the Affymetrix GeneChip Fluidics Station and scanned using an Affymetrix GeneChip Scanner 3000. Quality control analysis and data analysis produced.CEL image and.CHP image files for analysis using Affymetrix GeneChip Operating Software. Agilent GeneSpring GX 12 software was used to obtain datasets and perform statistical analyses; data normalisation was carried out using the MAS-5 algorithm and data baseline transformation to the mean of all samples. Data are deposited in Gene Ontology Omnibus (GEO, https://www.ncbi.nlm.nih.gov/geo/) and identified by the accession number: GSE132397.

### Genomic analysis

Normalised datasets generated by microarray analysis were analysed using ConsensusPathDB, Ingenuity IPA (Qiagen) and String V10.5 ^29,30^. Agilent GeneSpring GX 12 was used to assemble Affymetrix data and generate hierarchical clustering data and gene lists, from which astroglial genes were identified using multiple published datasets^31–33^. Lithium-responsive astroglial genes associated with BD were then determined using combined datasets from BDgene and DISGENET (V5.0)^34,35^. Gene Ontology (GO) sets were generated using ConsensusPathDB and String V10.5.

### SPIED/CMAP analysis

As previously described^36^, SPIED (Searchable Platform-Independent Expression Database) and CMAP (Connectivity MAP) were used to identify small molecules that are predicted to have the same gene signatures as the lithium-responsive astrglial genes and BD-associated genes identified by the genomic analysis described above. In brief, the expression profiles of lithium-responsive astroglial genes and those associated with BD were uploaded onto the SPIED database (http://spied.org.uk/cgi-bin/HGNC-SPIEDXXX.cgi) and interrogated against the CMAP initiative (https://clue.io/cmap), a database for over a thousand drug treatments, to identify drugs with gene expression profiles that correlate positively with our databases^37,38^. In this way, small bioactive molecules were identified that have the potential to mimic the effects of lithium on astrocytes and correlate with BD-associated genes; two of these drugs, Pioglitazone (1 mM) and 3-*O*-Epicatechin (3 µM) were validated in organotypic optic nerve cultures, as described above for lithium.

## Results

### Lithium induces astrocyte morphogenesis and proliferation

**Fig. 1:**
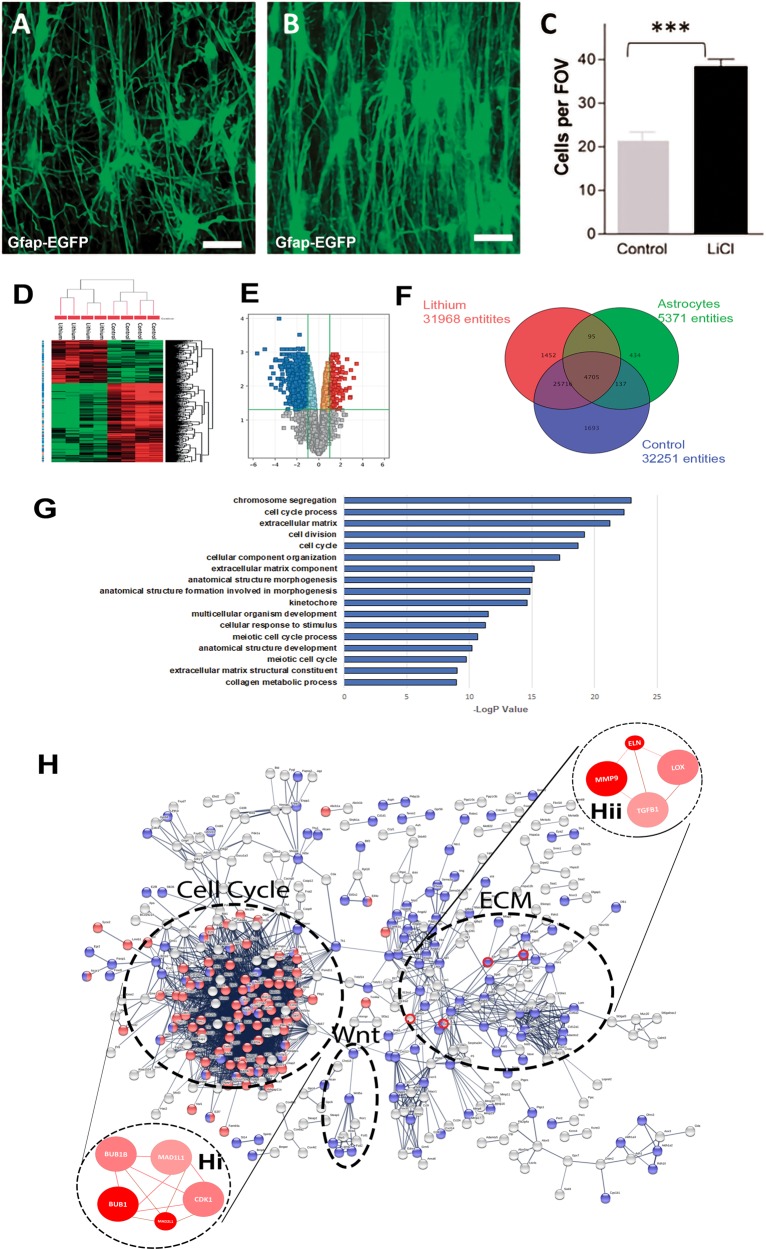
Identification of regulatory mechanisms of lithium in controlling astrocyte morphogenesis and proliferation. The effects of lithium on astrocytes were examined ex vivo in organotypic cultures of optic nerves from 5- to 6-week-old transgenic mice in which the astroglial gene glial fibrillary acidic protein (GFAP) drives the expression of enhanced green fluorescent protein (EGFP), to identify astrocytes. Confocal images of whole mounts of optic nerves maintained in culture for 3 days in control medium (**a**), or medium containing lithium (**b**). Compared to controls (**a**), lithium induced the formation of highly polarised astrocytes that formed a dense palisade traversing the nerve (**b**), Scale bars = 50 μm. **c** Graph of cell counts taken from a constant field of view (FOV, 200 µm x 200 µm) illustrating lithium doubled the number of astrocytes in the optic nerve; data are mean ± SEM (*n* ≥ 6 nerves in each treatment group), **p < 0.01, ***p < 0.001, Student’s *t* test. Microarray analysis using Affymetrix GeneChip Mouse Genome 430 2.0 was performed on optic nerves from 5- to 6-week-old C57/BL10 mice maintained ex vivo in organotypic cultures for 3 days in control medium or medium containing lithium. **d** Unsupervised hierarchical clustering of gene expression changes induced by lithium compared to controls, where red and green indicate up- and downregulated genes, respectively. **e** Volcano plot analysis was used to identify statistically represented genes with a false discovery rate (FDR) < 0.05 and an absolute fold change (FC) > 2.0. **f** Lithium responsive astroglial genes (4705 entities) were determined by interrogating the optic nerve gene datasets against the gene sets for astrocytes^31–33^. **g** GO analysis of astroglial genes altered by lithium identified cell cycle and extracellular matrix (ECM) as the major biological processes regulated by lithium. **h** Neighbourhood-based entity set analysis (NEST) of cell cycle genes (hi) and ECM genes (hii) identified by String analysis of predicted protein–protein interactions (circled in red). NEST analysis of ECM genes (hi, hii) identified lysyl oxidase (LOX), Elastin (Eln), Metalloprotienase 9 (Mmp9) and transforming growth factor beta 1 (Tgfb1) as major astroglial targets of lithium (dark and light red nodes have significant enrichment levels (*p* *<* 0.0001), whilst thickness of connection lines represent the number of shared genes across nodes, and colour shade represents the number of shared input genes)

Astrocytes are essential for WM function and integrity^24^, and are a potential target for the beneficial effects of lithium on WM in BD^25^. To examine the effects of lithium on WM astrocytes directly, we used a mouse optic nerve ex vivo preparation^27^, which was adapted so that the retina and optic nerve are maintained intact and kept alive in organotypic culture, in control medium or medium plus lithium. Optic nerves from adult GFAP*-*EGFP transgenic reporter mice were used^26^ to enable unequivocal identification of astrocytes. High resolution confocal microscopic examination of astrocyte three-dimensional morphology in whole-mounts of the intact nerve demonstrated that lithium has a striking effect on astrocytes, increasing their numbers and dramatically altering their morphology in all the preparations analysed (*n* = 6 per experimental group, in triplicate) (Fig. 1). In untreated cultures, mouse optic nerve astrocytes have a typical stellate morphology, with processes extending in all directions from a centrally located cell body (Fig. 1a). In marked contrast, lithium induced a highly polarised morphology in astrocytes (Fig. 1b) and doubled their numbers compared to controls (Fig. 1c; *p* < 0.001 unpaired *t*-test). Astrocyte processes characteristically bear fine collaterals and spines and have discrete process territorial domains in control nerves (Fig. 1a)^39^. In lithium, astrocytes form clones of densely packed cells that extend long smooth primary processes that rarely branched to form an astroglial palisade (Fig. 1b), typical of reactive astrogliosis^40^. The results demonstrate that lithium induces astrocyte proliferation and morphogenesis.

### LOX is a novel lithium-responsive astroglial gene

To elucidate the mechanism of action by which lithium induces the profound changes in astrocytes, we performed a microarray analysis to compare the gene expression profiles of optic nerves treated with lithium compared to controls and interrogated these against the gene sets for astrocytes generated by Barres and colleagues^31,41^. Hierarchical clustering demonstrated that entities were most closely related within experimental groups (Fig. 1d). Over 4000 astrocyte genes were differentially expressed in lithium-treated and control optic nerves (Fig. 1f) and these data were analysed by volcano plot and filtered for FC ≥ 2.0 and absolute value *E* ≥ 200 to identify the most significant lithium-responsive astrocyte genes (Fig. 1e; *p* < 0.05, moderated *t*-test and Benjamini–Hoechberg FDR). Unbiased IPA analysis was performed to identify the major lithium-responsive astrocyte genes (Supplementary Table 1). A key novel finding is that the most altered astrocyte gene in lithium was *LOX* (lysyl oxidase), which plays a critical role in remodelling of the extracellular matrix (ECM) and cell growth^42^, but has not previously been reported to have an important role in astrocyte remodelling or to be a target of lithium. Notably, lithium downregulated *Gas1* (growth arrest-specific 1) and *Il13ra1* (Interleukin 13 receptor, alpha 1), which are principle genes in reactive astrogliosis^41^, whilst lithium upregulated *Fstl1* (Follistatin-like 1), which inhibits bone morphometric protein (BMP) signalling and reactive astrogliosis mediated through STAT3 signalling^20,43^. Overall, the most lithium-responsive astroglial genes are associated with inhibition of reactive astrogliosis, whereas the most upregulated lithium-responsive astroglial genes are associated with astrocyte morphogenesis, proliferation and cell-cell interactions (Supplementary Table 1), indicating these effects on astrocytes are central to the positive therapeutic outcomes of lithium treatment.

### Cell cycle and ECM remodelling are key lithium-responsive astroglial networks

Transcriptomic analysis identified 1084 lithium-responsive astroglial genes and network analysis was performed on this dataset to identify the key astroglial signalling pathways that are regulated by lithium, using the ConsensusPathDB database^29^. Consistent with data presented above, the top functional categories were associated with the cell cycle, morphogenesis/development and ECM reorganisation, reinforcing the importance of these pathways in the observed effects of lithium on astrocytes (Fig. 1g). String V.10.5 Network Visualisation and Gene Ontology (GO) analysis was used to determine the astroglial signalling-to-transcriptional interactions that were induced by lithium (Fig. 1h). The most prominent transcriptional node was associated with the control of cell cycle, with *Bub1b* central to these actions (Fig. 1hi). The second most prominent node was associated with ECM remodelling, with *LOX* at its core (Fig. 1hii), together with its up- and downstream targets, *Tgfb1* (transforming growth factor β1), *Mmp9* (Metalloprotease 9) and *Eln* (Elastin). Interestingly, Wnt signalling was also identified as a lithium-responsive astroglial network and has recently been implicated in BD, schizophrenia and AD^44,45^. The results support LOX and ECM remodelling as being central to the morphogenic and proliferative effects of lithium on astrocytes.

### LOX is a novel astroglial gene associated with neuropsychiatric disorders

**Fig. 2:**
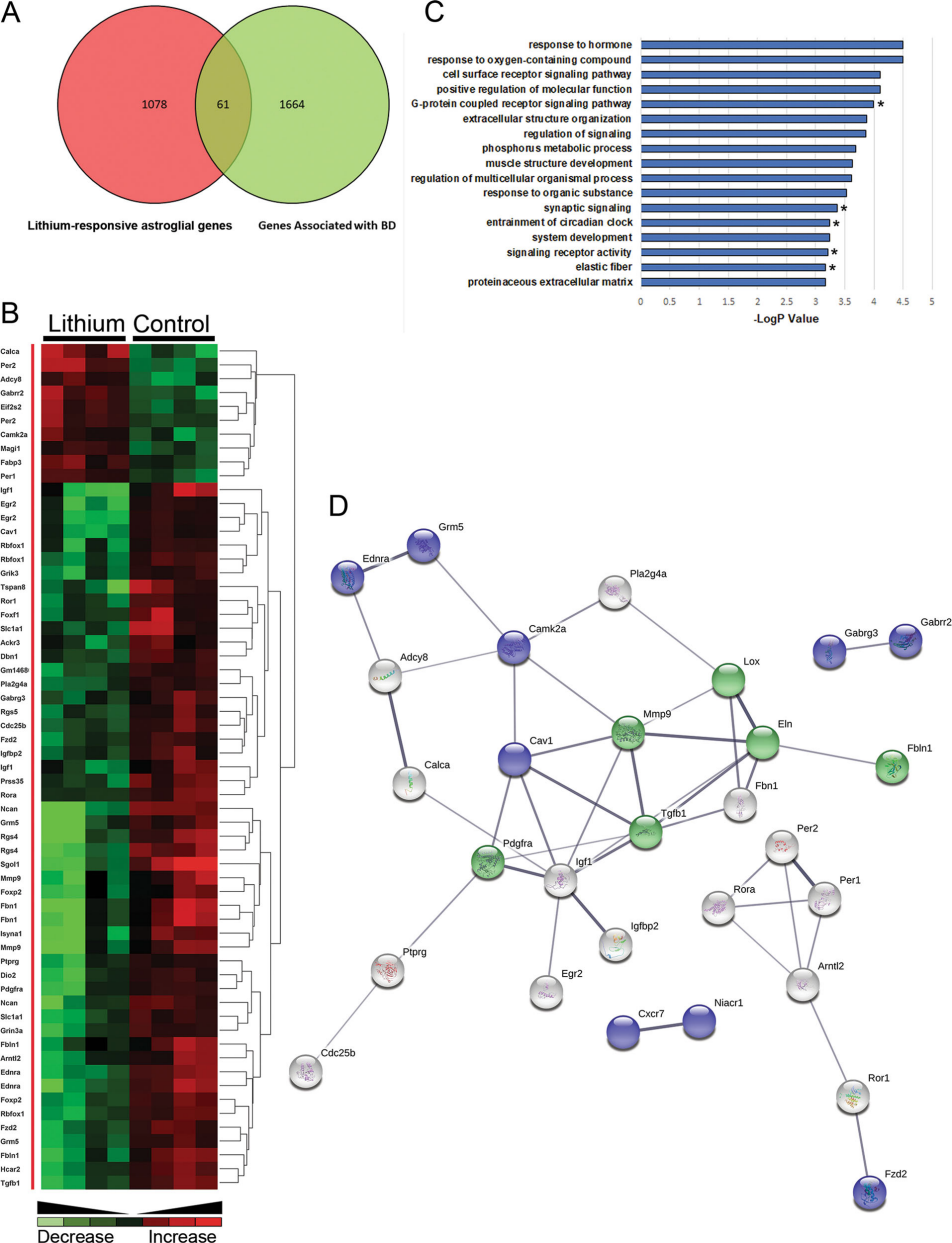
Lithium-responsive astroglial genes associated with bipolar disorder (BD). **a** Interrogation of lithium-responsive astroglial genes against BD databases DISGENET(5.0) and BDGene^34,35^ identified 61 potential outcome markers that are present in both groups. **b** Unsupervised hierarchical clustering illustrates the overriding effect of lithium to downregulate BD-associated astroglial genes. **c** GO analysis identifies receptor signalling and ECM organisation as major astroglial biological processes regulated by lithium and associated with BD; *Tgfb1* was found in all the GO terms apart from the five indicated with asterisks. **d** NEST analysis of lithium-responsive astroglial genes associated with BD predicted a major interaction between LOX and Elastin with TGFβ1 and MMP9 as the mechanism for astroglial ECM regulation and potential surrogate markers in BD (PPI enrichment *p* = 9.55e−15 *p* < 0.001; coloured nodes represent top biological pathways: green = ECM organisation (*p* < 0.001), blue = G-protein couple receptor signalling (GPCR) pathways (*p* < 0.001)

**Fig. 3:**
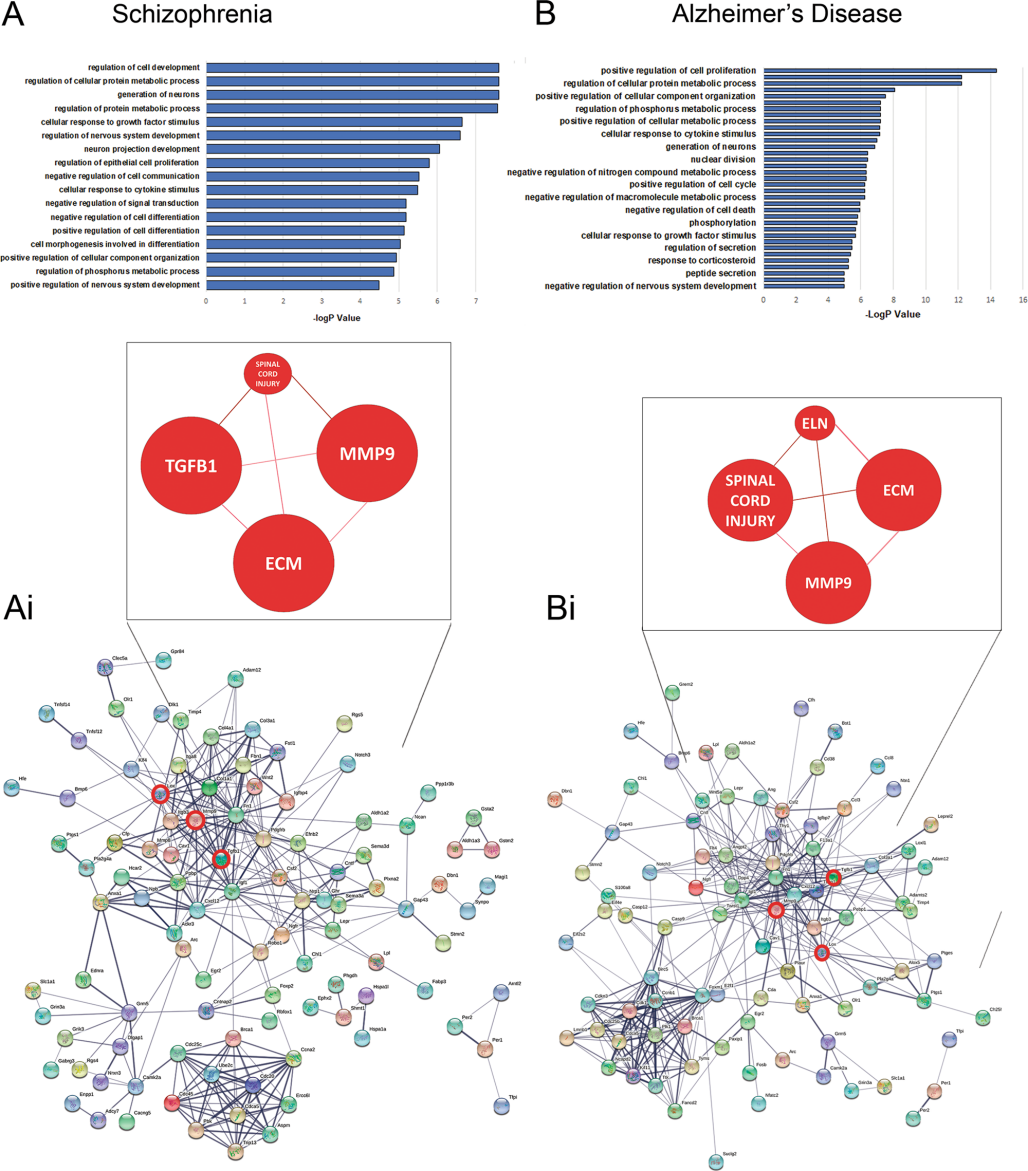
Identification of lithium-responsive genes associated with schizophrenia and Alzheimer’s disease. Lithium-responsive astroglial genes were interrogated to identify novel associations within the disease specific databases for schizophrenia and Alzheimer’s disease. GO analysis representing the biological pathways statistically altered in schizophrenia (**a**) and Alzheimer’s disease (**b**). Protein–protein prediction analysis and NEST analysis identifies MMP9, LOX and TGFB1 at the core of the networks for schizophrenia (ai) and Alzherimer’s disease (bi). (PPI enrichment *p* < 1.0e−16)

To better understand the therapeutic actions of lithium, we interrogated our unique database of lithium-responsive astroglial genes against the DISGENET(5.0) database and the well-characterised datasets for BD (BDGene; Fig. 2), as well as schizophrenia and AD (Fig. 3). We identified 61 lithium-responsive astroglial genes in the BDGene dataset^34,35^ (Fig. 2a), many of which are have defined associations with BD (Fig. 2b), such as Wnt signalling (Fzd2), neurocan, MMP9 and IGF-I^44,46–48^. To determine the potential therapeutic effects of lithium on astrocytes in BD, the functional classification of this new dataset was interrogated further using the ConsensusPathDB database^29^. The main GO terms were those associated with receptor signalling and ECM organisation (Fig. 2c). Neighbourhood-based entity set (NEST) analysis of the lithium-responsive astroglial BD susceptibility genes identified Tgfb1 as most strongly associated, together with Elastin (Eln1), which is the direct ECM target of LOX^49,50^. Furthermore, STRING analysis^30^ of the BD susceptible genes revealed that LOX and elastin participate in a major network with Tgfb1 and Mmp9 to regulate the ECM (Fig. 2d). Since lithium is also a therapy in schizophrenia and AD, we interrogated the lithium-responsive astroglial gene database against DISGENET (5.0) and the SZDB database (Fig. 3)^34,51,52^. We identified 179 entities associated with schizophrenia and147 entities associated with AD, and GO analysis determined the key biological astroglial pathways as regulation of cell proliferation and differentiation (Fig. 3ai, bi). Notably, network analysis placed Lox, Mmp9 and Tgfb1 at the core of the astroglial networks associated with AD (Fig. 3aii) and schizophrenia (Fig. 3bii). These findings identify LOX and ECM remodelling as being key to the mechanism of action of lithium on astrocytes and as putative surrogate outcome markers in BD and other neuropsychiatric disorders^47,53^.

### SPIED/CMAP identifies novel drugs for regulating astroglial function

Pharmacogenetics is an area of substantial growth in seeking new treatments for neurodegenerative and neuropsychiatric diseases^1^. We used this approach to identify potential therapies that may target the lithium-responsive astroglial pathways identified above. As previously described^36^, SPIED/CMAP meta-analysis was performed on ‘connectivity maps’ to identify small bioactive molecules that could be employed to generate the same transcriptional signature as lithium-responsive ‘astrocyte-modifying’ drugs (Table 1a), and applied to BD (Table 1b), Schizophrenia (Table 1c) and AD (Table 1d). Notably, the top ‘astrocyte-’ and ‘BD-modifying’ drugs act via PPAR-γ, namely piogliatazone (top ‘astrocyte-modifying’ drug, Table 1a) and betulinic acid (top ‘BD-modifying’ drug, Table 1b), both of which are used in the treatment of type-II diabetes, and in the case of piogliatazone has shown promising results for its anti-depressant activity in clinical trials^54,55^. Moreover, the PPAR-γ ligand 15-delta_prostaglandin_J2 was present as an astrocyte-, BD- and schizophrenia-modifying agent (Table 1a–c), further emphasising the potential importance of PPAR-γ in reactive astrogliosis and as a therapeutic target in neurodegenerative diseases^56–58^. The remaining drugs were specific for each disorder, with the exception of the flavonoids acacetin, (+/−)-catechin and quercetin, which were identified as astrocyte-, BD- and AD-modifying agents (Table 1a–d); significantly, flavonoids have mood stabilising properties and are protective against cognitive loss^59,60^. In addition, antipsychotics and cholinergics were prominent in the top schizophrenia-and AD-modifying drugs, suggesting astrocytes may be targets of these drugs in these diseases.

**Table 1:** Top-ranked small molecules identified from SPIED/CMAP analysis of lithium-responsive astrocyte genes that are predicted to be associated with (A) astrocytes, (B) bipolar disorder (BD), (C) schizophrenia and (D) Alzheimer’s diseases (AD)

Compound	Correl	Significance	Actions
(A) ‘Astrocyte-modifying’ agents			
**Pioglitazone**	**0.47**	**4.83**	**PPAR-gamma**
MG-262	0.33	4.66	Proteasome inhibitor
Semustine	0.35	4.44	Antineoplastic
**15-delta Prostaglandin J2**	**0.3**	**4.31**	**PPAR-gamma**
Econazole	0.39	4.23	Antifungal
Lomustine	0.33	4.19	Antineoplastic
Gossypol	0.34	4.13	Antineoplastic
**Acacetin**	**0.41**	**4.04**	**Flavonoid**
Parthenolide	0.32	4.03	Anti-inflammatory
Puromycin	0.31	3.98	Anti-neoplastic
(B) ‘BD-modifying’ agents			
**Betulin**	**0.99**	**4.59**	**PPAR-gamma**
Esculetin	0.96	3.27	Antineoplastic
Rottlerin	0.82	2.82	Antineoplastic
**Gliquidone**	**0.87**	**2.68**	**PPAR-gamma**
**(+/−)-Catechin**	**0.96**	**2.67**	**Flavonoid**
Butoconazole	0.99	2.58	Antifungal
Tiaprofenic acid	0.85	2.52	Antiinflammatory
**15-delta Prostaglandin J2**	**0.79**	**2.41**	**PPARg**
Celecoxib	0.98	2.32	Anti-inflammatory
Isradipine	0.82	2.31	Calcium channel blocker
(C) ‘Schizophrenia-modifying’ agents			
2,6-dimethylpiperidine	0.96	4.35	Anti-convulsant
Doxycycline	0.86	4.08	Antibiotic
Chlorprothixene	0.86	3.47	Antipsychotic
Sulpiride	0.79	3.39	Antipsychotic
W-13	0.6	3.33	Anti-calmodulin agent
Hycanthone	0.59	3.31	Anti-schistosomal
Lansoprazole	0.87	3.28	Proton pump inhibitor
Ethacrynic Acid	0.58	3.1	Loop diuretic
**15-delta Prostaglandin J2**	**0.47**	**2.92**	**PPAR-gamma**
Pregnenolone	0.62	2.91	Neuro-steroid
(D) ‘AD-modifying’ agents			
Edrophonium Chloride	0.77	3.21	Cholinesterase inhibitor
Oxybutynin	0.62	3.19	Anti-cholinergic
Metampicillin	0.55	3.02	Penicillin antibiotic
**Quercetin**	**0.8**	**2.87**	**Flavonoid**
Gossypol	0.61	2.75	Dehydrogenase inhibitor
Cephaeline	0.42	2.71	Emetic
Megestrol	0.69	2.68	Appetite stimulant
Captopril	0.96	2.68	ACE Inhibitor
Sulfamonomethoxine	0.49	2.64	Anti-bacterial
Spiperone	0.55	2.6	Antipsychotic

Small molecules are ranked according to the largest numbers of ‘target’ or ‘perturbed’ genes. Agents in bold are common to multiple datasets, notably flavonoids and drugs acting on PPAR-γ

### Drugs identified by pharmacogenomics have profound effects on astrocytes

**Fig. 4:**
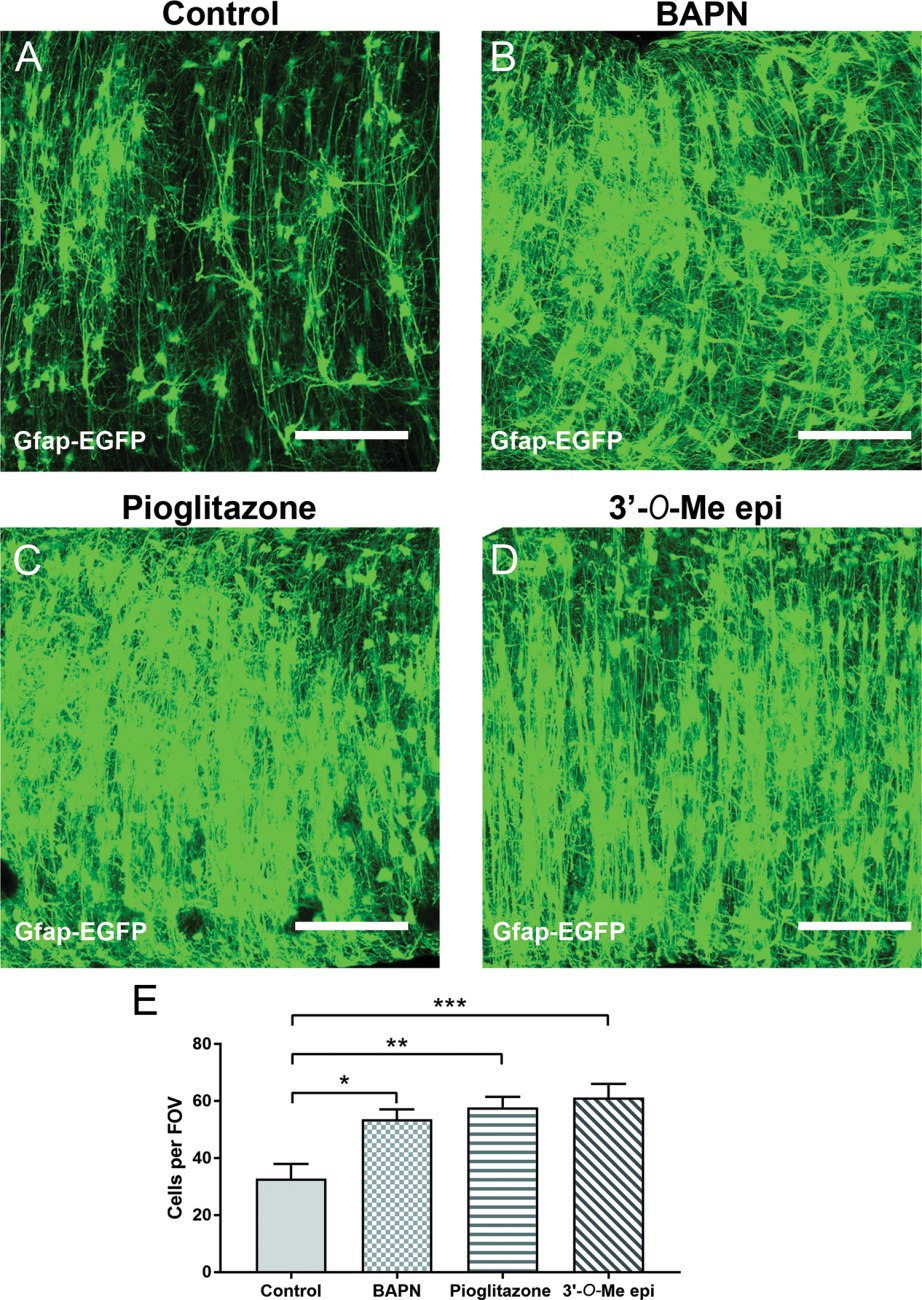
Drugs identified by SPIED/CMAP analysis mimic the effects of lithium on astrocytes. Inhibition of LOX was identified as the top most lithium-responsive gene (Supplementary Table 1) and SPIED/CMAP analysis of lithium-responsive astroglial genes associated with BD identified PPAR-γ agonists and flavonoids as small bioactive molecules with a high degree of correlation with Lithium. To validate these findings, the effects of the LOX inhibitor BAPN, the PPAR-γ agonist Pioglitazone and 3′-*O*-MethylEpicatechin were tested ex vivo in organotypic cultures of optic nerves from 5- to 6-week-old GFAP-EGFP transgenic mice to identify astrocytes. **a**–**d** Confocal images of whole mounts of optic nerves maintained in culture for 3 days in control medium (**a**), or medium containing BAPN (**b**), Pioglitazone (**c**) or 3′-*O*-MethylEpicatechin (**d**) illustrate all three agents induced a profound increase in astrocytes with highly polarised morphologies; scale bars = 100 μm in all panels. **e** Graph of cell counts taken from a constant field of view (FOV, 200 µm × 200 µm); data are mean ± SEM (*n* ≥ 6 nerves in each treatment group), **p < 0.01, ***p < 0.001, one-way ANOVA followed by Bonferroni post hoc test)

Our analyses identified LOX and PPAR-γ as primary astroglial targets of lithium that are relevant to BD and schizophrenia, whilst flavonoids have potential astrocyte-modifying effects that are relevant to BD and AD (Table 1). We therefore compared the actions of the LOX inhibitor β-aminopropionitrile (BAPN)^61,62^, the PPAR-γ ligand pioglitazone^63^ and the flavonoid 3’-*O-*Methylepicatechin^64^ on astrocytes in the optic nerve maintained *ex vivo* for 3 days (Fig. 4). Compared to controls (Fig. 4a), BAPN (Fig. 4b), pioglitazone (Fig. 4c) and 3’-*O-*Methylepicatechin (Fig. 4d) had striking morphogenic effects on astrocytes, inducing a highly polarised morphology and significantly increasing astrocyte cell numbers (Fig. 4e; ANOVA, followed by post hoc Bonferroni’s tests, *p* values indicated on graph). All three agents induced a dense palisade of astrocytes, equivalent to the actions of lithium (see Fig. 1), and unreservedly validate the astrocyte-modifying drugs with therapeutic potential identified by pharmacogenomics in Table 1.

## Discussion

Disruption of WM connectivity is a key feature of neuropsychiatric diseases^22,23^, and lithium has been shown to be beneficial for preserving WM in BD^23^. The underlying causes of WM disruption in BD are unresolved, but astrocytes are essential for WM structural and functional integrity^24^, and changes in astrocytes are implicated in BD, as well as other neuropsychiatric disorders and AD^5–8,11^. Here, using a combined neurobiological and pharmacogenomic approach, we demonstrate that lithium has striking effects on WM astrocytes and discover LOX and PPAR-γ as novel targets of lithium that profoundly regulate astrocyte proliferation and morphology (Supplementary Fig. 1). This study places astrocytes at the centre of the beneficial therapeutic effects of lithium and identifies promising surrogate biomarkers and potential disease-modifying drugs that target astrocytes in BD and other neuropathologies.

### Lithium induces a novel astrocyte phenotype

Lithium has a striking effect on astrocytes, inducing a unique morphological and genomic phenotype that is distinct from normal stellate astrocytes. The optic nerve is a model CNS tissue for studying glial cells *in situ*, because it can be isolated intact and does not contain neuronal somata, hence the transcriptome is made up almost entirely of glial cells^65^. Furthermore, white matter disruption is a key feature of neuropsychiatric diseases^22,23^, and lithium has been shown to be beneficial for preserving white matter integrity^23^. Astrocytes in lithium form a dense palisade, comparable to radial astrocytes in the developing CNS that are essential for supporting neuronal growth, axon targeting, dendrite arborisation and synaptogenesis^66^. Consistent with this, lithium upregulated Pacrg (Parkin Co-Regulated Gene), which is normally only abundant in radial glia^67^. Astrocytes in lithium are reminiscent of interlaminar astrocytes in the human and primate cortex that are increased in neuropsychiatric disorders^68^. Our results are consistent with evidence that astrocytes are modified by lithium treatment in neuropsychiatric disorders^13,14,16,25,69,70^. Moreover, genomic analyses demonstrate that lithium downregulates genes associated with reactive astrogliosis, identifying Fstl1 (Follistatin-like 1) as one of the top lithium-responsive astroglial genes, which acts via GSK3β-mediated BMP-STAT3 signalling to regulate reactive astrogliosis^43,71^. Reactive astrogliosis is used as an umbrella term for the morphological, physiological and transcriptional changes that astrocytes undergo in response to pathology^4,10^, stereotypically defined pathologically by the upregulation of GFAP, cellular hypertrophy, proliferation and glial scar formation. However, astroglial pathological changes are not uniform and are highly context-specific, ranging from subtle responses that are beneficial for neuroprotection and repair, to an extreme where reactive astrocytes are deleterious and form the glial scar^4,10^. Our results demonstrate that lithium induces an astrocyte morphological and genomic phenotype that is neuron-supportive and differs from astrocytes associated with glial scars, providing a potential mechanism by which lithium positively affects therapeutic outcomes in BD and other neuropathologies^11,19,72^.

### LOX is a novel astroglial therapeutic target

Genomic analyses identified the enzyme LOX as the most highly regulated lithium-responsive astroglial gene and this was validated using the irreversible LOX inhibitor BAPN, which mimicked the effects of lithium and demonstrates for the first time that LOX is a major regulator of astrocyte morphology and proliferation. In the CNS, LOX is expressed by both astrocytes and neurones and increased LOX activity is associated with decreased neurite outgrowth and plasticity^73^. Increased LOX is associated with pathological progression of ALS, where it is a potential biomarker^74^, and in AD LOX is implicated in plaque formation and colocalises with astrocytes associated with Aβ plaques^75^. In addition, there is evidence that LOX is upregulated in reactive astrocytes and that inhibition of LOX with BAPN improves recovery from spinal cord injury^76^. Furthermore, drugs that target LOX are already considered for cancer^77^. Ours is the first study that identifies LOX as an astroglial target of lithium and as a common factor and potential surrogate biomarker in BD, schizophrenia and AD.

LOX is a copper-dependent amine oxidase that is crucially involved in ECM synthesis and processing by catalysing the covalent cross-linking of collagens and elastin fibres that are responsible for ECM stability^42,78^. Our analyses placed LOX at the centre of ECM remodelling networks that are associated with BD, schizophrenia and AD, involving Elastin, which is the direct target of LOX, together with TGF-β1 and MMP9. The ECM has a critical role in brain plasticity and is known to be altered in BD and other neuropsychiatric disorders^53,79,80^. Moreover, TGF-β1 and MMP9 have been identified as potential biomarkers of BD disease progression and lithium responsiveness^47,81,82^. The interactions of TGF-β1 and MMP9 with LOX are complex and context specific, but TGF-β1 and MMP9 generally act to stimulate LOX activity and ECM stabilisation via elastin-collagen cross-linking^42,78^. Notably, TGF-β1 has arisen as a potential biomarker and therapy in BD, depression and AD^82,83^. Thus, TGF-β1 and LOX are critically linked to astroglial neuropathological responses and agents that act to inhibit LOX have considerable disease modifying potential.

### PPAR-γ is a major astroglial lithium-responsive pathway associated with BD

Drugs that act on PPAR-γ were identified by SPIED/CMAP analysis as potential astrocyte-modifying agents in BD and schizophrenia. We verified this using piogliatazone, which had a profound effect on astrocytes, equivalent to that observed for lithium and the LOX inhibitor BAPN. The activation of selective PPAR isotypes are implicated in reactive astrogliosis and represent new potential therapeutic targets in traumatic brain injuries and neurodegenerative diseases^58^. Moreover, pioglitazone is used in the treatment of type-II diabetes and has shown promising results for its anti-depressant activity in clinical trials^54,55,84,85^. PPAR-γ is one of a subfamily of nuclear receptors that form heterodimers with retinoid X receptors (RXRs) to modulate the transcription of its target genes, such as acyl-CoA oxidase. Lithium increases PPAR-γ^86^ and PPAR-γ has been shown to inhibit LOX^87^; these effects may involve GSK3β-Wnt signalling, which is implicated in neuropsychiatric disorders and was identified to interact with ECM remodelling in our network analysis. In addition, PPAR-γ agonists decrease TGF-β1 signalling leading to decreased fibrosis^88^, providing a further link with LOX and the actions of lithium (Supplementary Fig. 1). Interestingly, SPIED/CMAP analysis also identified flavonoids as astrocyte-modifying agents with therapeutic potential in BD and AD. We demonstrate for the first time that the flavonoid epicatechin directly regulates astrogliosis and morphogenesis. Epicatechin is flavonol rich in cocoa, grapes and green tea with pleiotropic roles in neuronal protection. This flavonol is of particular interested for its ability to cross the blood–brain barrier when metabolised to 3′*-O-*MethylEpicatechin and its protective effect has been investigated in many neuropathologies, including anxiety, mood disorder, as well as cognitive decline^59,60^. Recent studies have shown that epicatechin can transactivate PPAR-γ in vitro^64,89^ and this could explain the high correlation with Pioglitazone in our analysis. Our results clearly demonstrate that drugs targeting LOX and PPAR-γ mimic the effects of lithium on astrocytes and, therefore, targeting these pathways in astrocytes has considerable therapeutic potential in neuropsychiatric disorders.

## Summary and conclusions

In summary, our combined neurobiological and genomic analyses demonstrate astrocytes are direct targets of lithium treatment and idendtified novel lithium-responsive genes that are promising surrogate biomarkers in BD, schizophrenia and AD. Using a pharmacogenomic approach, we developed a comprehensive catalogue of small molecules that can be used to manipulate astrocytes with potential therapeutic benefits. The power of this approach is highlighted by our demonstration that drugs acting on LOX and PPAR-γ have profound effects on astrocytes and that the same effects were observed for the flavonoid epicatechin. In this regard, two of the BD-modifying drugs we identified are antifungal, which is noteworthy because there is a strong drive for repurposing drugs that have known combined antifungal and antipsychotic activity^90^. Furthermore, our SPIED/CMAP analysis predicts that further disease-modifying effects could be achieved by combinatorial approaches that target PPAR-γ and neurotransmitter signalling, such as the α-1 adrenergic receptor antagonist phenoxybenzamine^91^, and the calcium channel antagonist nifedipine, which has been used in a small cohort study with BD patients^92^. Nifedipine has also been shown to activate the PPAR-γ pathway and suppress atherosclerosis in an animal model^93^. In conclusion, our study establishes unequivocally that astrocytes are a target of lithium treatment and provides a robust framework for a mechanistic approach to identify new therapeutics for diverse neuropsychiatric and neurodegenerative diseases.

## Supplementary information

Supplementary Figure and Table Legends

Supplementary Table 1

Supplementary Figure 1
