# Supplementary material for: Astrocytes are direct cellular targets of lithium treatment: novel roles for lysyl oxidase and peroxisome-proliferator activated receptor-γ as astroglial targets of lithium
Source: Transl Psychiatry. 2019 Sep 2;9:211. doi: 10.1038/s41398-019-0542-2 (PMC6718419; doi:10.1038/s41398-019-0542-2)
Supplement: Supplementary file 1 — Supplementary Figure and Table Legends [file 41398_2019_542_MOESM1_ESM.docx]

**Supplementary Material**

**Supplementary Table 1.** Top-ranked astroglial genes altered by lithium.

**Supplementary Figure 1. Mechanisms of action of lithium on astrocytes.** Astrocytes treated with lithium had a highly polarised morphology with cells extending long smooth process to traverse the optic nerve. Genomic analysis identified the LOX as a novel target of lithium in astrocytes, interacting with TGFβ, MMP9 and Elastin to mediate extensive remodelling of the ECM and cellular growth. LOX activity increases collagen cross-linking to promote ECM stiffness and inhibit cellular growth and plasticity. Inhibition of LOX by lithium or BAPN decreases collagen cross-linking to promote ECM remodelling and stimulate astrocyte growth and plasticity. The actions of lithium and BAPN were mimicked by the PPARγ agonist Pioglitazone indicating PPARγ is a target of lithium and acts by inhibiting LOX activity in astrocytes to promote growth and plasticity. These molecular targets of lithium in astrocytes are potential surrogate markers in BD and targeting these pathways in astrocytes has considerable therapeutic potential for regulating astroglial neuropathological responses.
