# Supplementary material for: Astrocytes are direct cellular targets of lithium treatment: novel roles for lysyl oxidase and peroxisome-proliferator activated receptor-γ as astroglial targets of lithium
Source: Transl Psychiatry. 2019 Sep 2;9:211. doi: 10.1038/s41398-019-0542-2 (PMC6718419; doi:10.1038/s41398-019-0542-2)
Supplement: Supplementary file 2 — Supplementary Table 1 [file 41398_2019_542_MOESM2_ESM.docx]

**Table 1** Top lithium-responsive astroglial genes identified by IPA

| **Symbol** | **Entrez Gene Name** | **p-value** | **Fold Change** |
| --- | --- | --- | --- |
| Lox | Lysyl oxidase | 0.001294 | -9.96 |
| Gas1 | Growth arrest-specific 1 | 5.34E-04 | -3.93 |
| Fstl1 | Follistatin-Like 1 | 7.47E-05 | -2.73 |
| Pacrg | PARK2 Co-Regulated | 3.38E-04 | 2.57 |
| Il13ra1 | Interleukin 13 Receptor Alpha 1 | 0.032208 | -2.43 |
| Alcam | Activated Leukocyte Cell Adhesion Molecule | 2.98E-05 | 2.42 |
| Cntf | Ciliary Neurotrophic Factor | 0.001198 | 2.31 |
| Klf4 | Kruppel-Like Factor 4 | 3.79E-04 | 2.27 |
| Per1 | Period Circadian Clock 1 | 0.001993 | 2.25 |
