# Supplementary figures and images for: Astrocytes are direct cellular targets of lithium treatment: novel roles for lysyl oxidase and peroxisome-proliferator activated receptor-γ as astroglial targets of lithium
Source: Transl Psychiatry. 2019 Sep 2;9:211. doi: 10.1038/s41398-019-0542-2 (PMC6718419; doi:10.1038/s41398-019-0542-2)

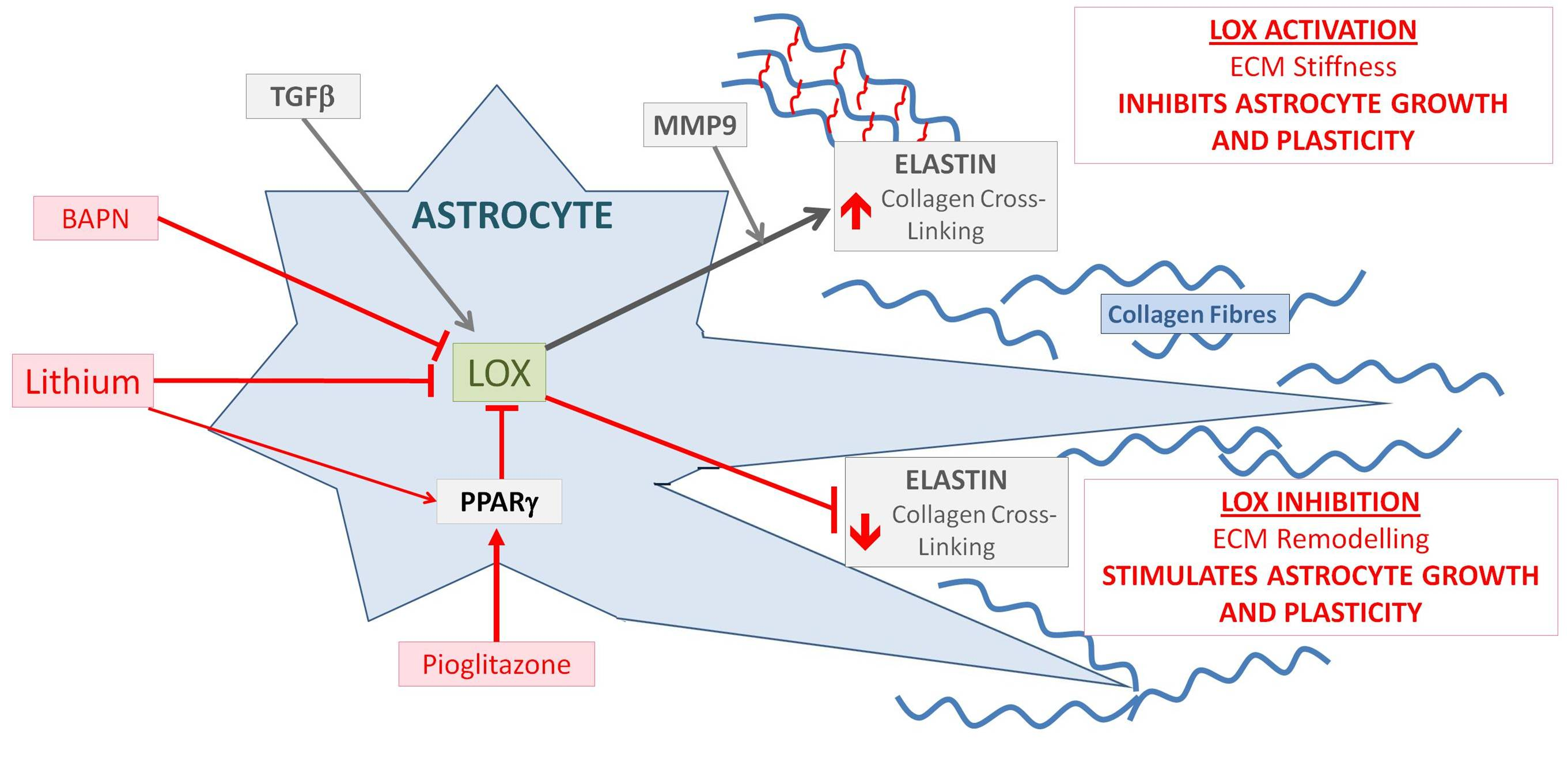

Supplement: Supplementary file 3 — Supplementary Figure 1 [file 41398_2019_542_MOESM3_ESM.tif]
